# Natural compounds from freshwater mussels disrupt fungal virulence determinants and influence fluconazole susceptibility in the presence of macrophages in *Cryptococcus neoformans*

**DOI:** 10.1128/spectrum.02841-23

**Published:** 2024-02-08

**Authors:** Davier Gutierrez-Gongora, Michael Woods, Ryan S. Prosser, Jennifer Geddes-McAlister

**Affiliations:** 1 Department of Molecular and Cellular Biology, University of Guelph, Guelph, Ontario, Canada; 2 School of Environmental Sciences, University of Guelph, Guelph, Ontario, Canada; The University of Iowa, Iowa City, Iowa, USA

**Keywords:** *Cryptococcus neoformans*, invertebrates, mussels, extracts, antifungals, virulence determinants, drug resistance, quantitative proteomics, One Health

## Abstract

*Cryptococcus neoformans* is a human fungal pathogen responsible for fatal infections, especially in patients with a depressed immune system. Overexposure to antifungal drugs due to prolonged treatment regimens and structure-similar applications in agriculture have weakened the efficacy of current antifungals in the clinic. The rapid evolution of antifungal resistance urges the discovery of new compounds that inhibit fungal virulence determinants, rather than directly killing the pathogen, as alternative strategies to overcome disease and reduce selective pressure toward resistance. Here, we evaluated the efficacy of freshwater mussel extracts (crude and clarified) against the production of well-defined virulence determinants (i.e., thermotolerance, melanin, capsule, and biofilm) and fluconazole resistance in *C. neoformans*. We demonstrated the extracts’ influence on fungal thermotolerance, capsule production, and biofilm formation, as well as susceptibility to fluconazole in the presence of macrophages. Additionally, we measured the inhibitory activity of extracts against commercial peptidases (family representatives of cryptococcal orthologs) related to fungal virulence determinants and fluconazole resistance, and integrated these phenotypic findings with quantitative proteomics profiling. Our approach defined distinct signatures of each treatment and validated a new mechanism of anti-virulence action toward the polysaccharide capsule from a selected extract following fractionation. By understanding the mechanisms driving the antifungal activity of mussels, we may develop innovative treatment options to overcome fungal infections and promote susceptibility to fluconazole in resistant strains.

**IMPORTANCE:**

As the prevalence and severity of global fungal infections rise, along with an increasing incidence of antifungal resistance, new strategies to combat fungal pathogens and overcome resistance are urgently needed. Critically, our current methods to overcome fungal infections are limited and drive the evolution of resistance forward; however, an anti-virulence approach to disarm virulence factors of the pathogen and promote host cell clearance is promising. Here, we explore the efficacy of natural compounds derived from freshwater mussels against classical fungal virulence determinants, including thermotolerance, capsule production, stress response, and biofilm formation. We integrate our phenotypic discoveries with state-of-the-art mass spectrometry-based proteomics to identify mechanistic drivers of these antifungal properties and propose innovative avenues to reduce infection and support the treatment of resistant strains.

## INTRODUCTION

Fungal infections pose a significant threat to human health worldwide. *Cryptococcus neoformans* is a human fungal pathogen found ubiquitously within the environment and of particular concern due to its ability to cause severe infections in immunocompromised individuals, especially HIV/AIDS patients (1). With a 73% worldwide mortality rate (including a 12% mortality rate in the United States alone), this yeast is one of the deadliest fungal pathogens (2, 3). Currently, three classes of antifungal drugs are effective at treating cryptococcal infections (i.e., azoles, polyenes, and flucytosine); however, their overuse in clinical settings has produced an emergence of drug-resistant fungi that limit drug efficacy (4). Notably, some of these antifungals (e.g., azoles) are also used in agricultural venues where environmental microorganisms, such as *Aspergillus fumigatus* and *C. neoformans*, may trigger resistance mechanisms outside the host, reducing therapeutic options in the clinic (5–7).

Combining these overlapping areas, fungal infections have turned into a One Health problem (i.e., the interconnectivity across environmental, animal, and human health), highlighting the urgent need to explore alternative therapeutic strategies, such as targeting fungal virulence determinants instead of cell growth to reduce pathogen burden and promote immune system clearance (8). The virulence determinants of *C. neoformans* play crucial roles in fungal pathogenicity and contribute to the severity of infection (9). One of the most important is the polysaccharide capsule, which is comprised mainly of glucuronoxylomannan (GXM) and protects the fungus from desiccation within the natural environment and the phagocytic action of the host immune system (10). Inhibiting capsule formation or disrupting its structure increases the vulnerability of the fungus to an immune attack and enhances the efficacy of antifungal treatments (9, 11). Melanin is another critical virulence determinant, a dark pigment that protects against environmental stresses and antifungal drugs (12). Targeting the melanin synthesis pathway could increase the susceptibility of *C. neoformans* to antifungal agents and reduce its ability to cause disease.

The secretion of multiple enzymes (e.g., urease, phospholipases, and peptidases) by *C. neoformans* also contributes to fungal virulence by damaging host cells and tissues and promoting fungal survival and dissemination to the brain (13, 14). Inhibition of these enzymes is a potential strategy to reduce fungal damage and limit spread within the host (15). Thermotolerance mechanisms to human temperature (37°C) are also considered an important virulence factor that allows the pathogen to grow and disseminate within the host when other microorganisms cannot (16). Moreover, although not considered a traditional virulence factor, the cryptococcal biofilm constitutes a common defense mechanism against host and antifungal action by decreasing their effectiveness (17). The inhibition of biofilm formation or promotion of biofilm disruption are desired properties in discovery pipelines (18). Taken together, by disrupting these key virulence determinants, we aim to increase the efficacy of existing antifungal agents or reduce the severity of infections caused by *C. neoformans* with potential extrapolation to other fungal pathogens using similar mechanisms of virulence without disruption to the normal human microbiota (19, 20).

The specificity and toxicity of novel agents targeting fungal pathogens must be carefully prioritized, given the shared structures and metabolic pathways between fungal pathogens and their human hosts (21). In this context, natural compounds provide promising antifungal candidates due to selection over evolutionary timescales as effective defense mechanisms against multiple pathogens without causing adverse effects to the host (22, 23). Invertebrates are one of the most diverse groups of organisms on the planet, possessing an effective innate immune system against multiple pathogens with minimal cytotoxic effect on mammalian cells (24–27). Critically, invertebrates represent a promising reservoir for novel compound discovery with antifungal properties, and specifically, the efficacy of extracts (crude and clarified) from freshwater and terrestrial mollusks have proven diverse antifungal activity against *C. neoformans* (28, 29).

In the current study, we assessed the antifungal properties of two freshwater mussel species against *C. neoformans*, focusing on extract impact on fungal virulence determinants and susceptibility to fluconazole. We prepared an aqueous extract from *Dreissena polymorpha* and segmented aqueous extracts from *Lasmigona costata*. Upon fungal treatment with the extracts, we observed significant targeted effects toward thermotolerance, biofilm formation, and capsule production, as well as an influence on susceptibility to fluconazole in the presence of macrophages. These observations were dependent upon the extract source and clarification process. Additionally, we performed inhibitory activity assays against multiple commercial peptidases from *Saccharomyces cerevisiae* (family representatives of cryptococcal orthologs) related to virulence determinant production. We also demonstrated correlative findings along with complementation of the phenotypic assays with molecular profiling of each extract using mass spectrometry-based proteomics (30, 31). Integration of these data sets defined core and unique protein signatures across the mussel extracts, followed by the validation of new mechanisms of anti-virulence action against *C. neoformans*. Further, fractionation of a selected extract revealed specific activity supported by validation of compound class and *in vitro* inhibition, as well as reduced capsule production. Overall, our findings stress the importance of evaluating and quantifying the efficacy of natural sources to fight globally devastating fungal pathogens. By understanding mechanisms driving the antifungal activity of mussels, we may develop novel treatments for fungal infections and decrease resistance to conventional antifungal drugs.

## MATERIALS AND METHODS

### Protein extraction

Proteins were extracted from two species of freshwater mussels, *D. polymorpha* (zebra mussel) and *L. costata* (fluted shell mussel). *Dreissena polymorpha* were collected from Sand Lake in Ontario, and *L. costata* from the Grand River in Ontario. Mussel tissue extraction was performed as previously described with some variations (32). Briefly, 20–25 g of tissue from mussels was cut, pooled, flash frozen, and ground with a mortar and pestle. For *L. costata*, organs that receive the greatest exposure to the environment outside the valves were dissected into gill, foot, and mantle before tissue homogenization. The resulting powder was resuspended in Milli-Q water using a 1:2 ratio (wt:vol) with metal beads (1 g/mL) and further disrupted using a bullet blender at 1200 rpm for 5 min at 4°C. Next, samples were centrifuged at 12,000 × *g* for 20 min at 4°C obtaining a crude extract (i.e., non-clarified). Clarified extracts were obtained using thermal precipitation at 60°C for 30 min and centrifuged at 15,000 × *g* for 45 min at 4°C as previously described (29). All samples were filtered with 0.22 µm membranes, aliquoted, and stored at −20°C until use. The bicinchoninic acid assay (BCA) was used to measure the total protein concentration for each extract (33).

### Strains and growth conditions

*C. neoformans* variety *grubii* strain H99 (wild type) was used for all cultures related to virulence factor assays. Fungal strains were routinely kept on yeast peptone dextrose (YPD) agar (1% yeast extract, 2% Bacto-peptone, 2% D-glucose, and 2% agar) and stored at 4°C. The clinical (CL) fluconazole-resistant (64 µg/mL) *C. neoformans* var. *grubii* H99 strain was generously donated by Dr. Jennifer Tenor and Dr. John Perfect (Duke University) and maintained on YPD plates supplemented with 64 µg/mL fluconazole and 34 µg/mL chloramphenicol.

Overnight cultures of *C. neoformans* were washed and resuspended in Yeast Nitrogen Base (YNB) to a concentration of 10^5^ cells/mL. Next, to assess the effect of mussel extracts (crude and clarified) on the growth of *C. neoformans* at 37°C and 30°C, 10 µL of an extract serial dilution series was mixed with 190 µL of fungal cells (previously resuspended in YNB) in a 96-well plate. Growth curves were measured using a plate reader (Synergy-H1, Biotek) at 200 rpm for 60 h, followed by optical density (OD_600nm_) readings every 15 min. All experiments were performed in five biological and two technical replicates.

### Capsule production

Overnight cultures of *C. neoformans* H99 from YPD were sub-cultured in YNB and incubated overnight at 30°C with 200 rpm shaking. Production of polysaccharide capsule was induced as previously described (29). Briefly, 10^5^ cells/mL were cultured in 5 mL of low iron media (LIM; 0.5% L-asparagine, 0.4% HEPES, 0.04% K_2_HPO_4_, 0.008% MgSO_4_ · 7H_2_O, 0.2% NaHCO_3_, and 0.025% CaCl_2_ · 2H_2_O) and incubated for 72 h at 37°C. Visualization of cells was performed by mixing cells with India ink using a 1:1 ratio on microscope slides and visualizing with a Differential interference contrast (DIC) microscope and a 63× oil objective. Capsule production was quantified using a ratio of total cell size (with capsule) to cell size (without capsule). All measurements were obtained using three biological replicates with 50–60 cells assessed per condition, and the experiment was performed in technical duplicate.

For E-64 treatment to validate the proteome findings, *C. neoformans* H99 cells were prepared as described and treated with E-64, a general cysteine-like peptide inhibitor (34), to confirm the involvement of cysteine-like peptidases (e.g., CNAG_05601) in capsule production (35). Capsule production was visualized and quantified as stated above.

### Melanin production

Melanin production was induced as described previously (29). Briefly, overnight *C. neoformans* H99 cultures were sub-cultured in YNB overnight and incubated overnight at 30°C and 200 rpm. Afterward, the cells were collected by centrifugation and resuspended in phosphate-buffered saline (PBS) to reach a final concentration of 10^6^ cells/mL. Treated cells were incubated with 20 µL of mussel extracts (crude or clarified) for 4 h, serially diluted and spread on L-3,4-dihydroxyphenyl-alanine (L-DOPA) agar plate [1.4% agar, 13 mM glycine, 30 mM KH_2_PO_4_, 10 mM MgSO_4_ · 7H_2_O, 5 mM glucose, 2.8 µM thiamine, 1 mM L-DOPA (Sigma)]. Plates were statically incubated for 48–72 h at 37°C, with pictures taken every 24 h under standardized conditions in a photographic chamber. To quantify melanin production, photographs were analyzed as previously described (36). All measurements were obtained using six biological and two technical replicates.

### Biofilm formation

Biofilm formation was induced using a previously described protocol (29). Briefly, overnight *C. neoformans* H99 cultures in YNB were resuspended in Dulbecco’s modified Eagle’s medium (DMEM) (Corning). To potentially reduce effects on growth, a concentration of 10^6^ cells/mL, 10 times higher than for growth, was used. Next, 285 µL of fungal cells combined with 15 µL of protein extracts or PBS (control) was transferred into individual wells of sterile, polystyrene, flat-bottom, 24-well microtiter plates (Corning), and statically incubated at 37°C for 48 h. After the incubation period, the supernatant from each well was removed and washed two times with sterile water and air-dried for 10 min at room temperature.

For biofilm quantification, cells were washed two times, stained with crystal violet solution, and incubated at room temperature for 10 min (37). Next, each well was thoroughly washed, and biofilms were destained with 100% ethanol for 10 min at room temperature. Finally, the destained solution from each well was transferred to a new 96-well microtiter plate, and OD_550nm_ was measured using a plate reader (Synergy-H1, Biotek). All measurements were performed using four biological replicates, and the experiment was repeated in duplicate.

### Biofilm disruption

For biofilm disruption, wells with fungal cells only were prepared as described above with static incubation in DMEM at 37°C wrapped in aluminum foil for 24 h. At this time, 15 µL of mussel extract (crude and clarified) was added to each well to achieve a final volume of 300 µL and incubated for an additional 24 h at 37°C wrapped in aluminum foil. All measurements were performed using four biological replicates, and the experiment was repeated in duplicate.

### Osmotic and cell membrane stress assays

Osmotic and cell membrane stress were assessed using YPD agar plates supplemented with NaCl (1 M) and SDS (0.01%), respectively. In each case*, C. neoformans* cells were grown to mid-log phase in YPD at 30°C and 200 rpm, normalized to 10^6^ cells/mL, and incubated with each extract and/or fluconazole (8 µg/mL for H9) for 4 h at 30°C and 200 rpm. The cells were serially diluted 10-fold from 10^6^ to 10^1^ cells/mL, and 5 µL was spotted on each plate. YPD plates supplemented with the different stressors were statically incubated at 30°C and 37°C. Growth was followed by taking images every 24 h for a minimum of 72 h. Each experiment was performed using three biological and two technical replicates.

### Urease activity assay

To assess the effect of the extracts on secretory pathways, we evaluated urease activity as previously described with minor variations (38). Briefly, *C. neoformans* H99 cells were grown to mid-log phase in YPD at 30°C and 200 rpm, normalized to 10^6^ cells/mL, and incubated with each extract for 4 h at 30°C and 200 rpm. The cells were serially diluted 10-fold from 10^6^ to 10^1^ cells/mL, and 5 µL was spotted in Christensen’s Urea Agar (peptone 0.1%, glucose 0.1%, NaCl 0.5%, KH_2_PO_4_ 0.2%, Phenol red 0.0012%, urea 2%, and agar 1.5%) (39). Urea agar plates were statically incubated at 30°C and 37°C. Urease activity was followed by taking images at 24 h and measuring the ratio between the halos and the cells. Each experiment was performed using three biological and two technical replicates.

### Macrophage infection

To assess the susceptibility of the WT (H99) and CL strains against immortalized macrophages derived from BALB/c mice in the presence of the mussel extracts, the fungal cells were grown as previously described with some variations (40). Briefly, macrophages were normalized to 50,000 cells/mL using DMEM supplemented with penicillin and streptomycin and statically incubated at 37°C, CO_2_ 5% for 48 h. The *C. neoformans* strains were cultured in liquid YPD at 37°C and 200 rpm for 18 h and sub-cultured O/N in the same conditions. Cryptococcal cells were collected by centrifugation and washed two times with PBS. Using a hemocytometer, the cells were normalized to 1 × 10^6^ cells/mL in 1 mL of DMEM. Cryptococcal cells were opsonized by adding 1 µg of anti-GXM antibodies (1 µg/mL) and incubated for 1 h at 37°C and 5% CO_2_.

For infection, macrophages were gently washed with 1 mL of PBS and diluted in DMEM with *C. neoformans* (10^6^ cells/mL). After static incubation for 90 min at 37°C and 5% CO_2_, the cells were washed two times with PBS and diluted in DMEM with extracts (50 µL) and/or 64 µg/mL of fluconazole and incubated for 15 h at 37°C and 5% CO_2_. Macrophages were washed two times with PBS and incubated with 1.2% Triton X-100 for 10 min at room temperature. From each well, 1 mL was collected and serially diluted in PBS using a 10-fold series until 10 cells/mL was achieved. From each dilution, 100 µL was plated on YPD-agar plates, allowed to dry at room temperature for 10 min, and incubated at 30°C for 48 h. Colony-forming units (CFUs) were counted on each condition and dilution.

### Cytotoxicity assay

To analyze the cytotoxic effect of extracts against mammalian cells, we assessed the lactate dehydrogenase (LDH) activity of BALB/c macrophages as previously described (40). Extract (10 µL) was combined with 1 mL DMEM-containing macrophages using 24-well plates and, statically, incubated at 37°C, 5% CO_2_ for 4 h. Triton (1.2%) was added to the non-treated wells for total death and incubated at room temperature for 30 min. LDH substrate (NAD^+^) (Sigma-Aldrich, USA) and samples were mixed using a 1:1 ratio (vol:vol) in 96-well plates and incubated at room temperature for 20 min before adding a stopping solution (Sigma-Aldrich, USA). LDH activity was quantified by measuring OD_450nm_. Media only and Triton 1.2% were used as blanks for extract-containing wells and total death replicates. Each experiment was performed using three biological and two technical replicates.

### Fluconazole resistance

To assess the effect of mussel extracts on fluconazole resistance, cryptococcal cells were prepared as outlined above for growth assays. Each sample was mixed with the CL strain in the presence or absence of fluconazole (64 µg/mL) in 96-well plates wrapped in aluminum foil and incubated at 37°C and 200 rpm for 72 h. Growth was discontinuously followed at OD_600nm_ every 24 h. Optical density values on each condition were normalized against the corresponding control (i.e., cells with or without fluconazole).

### Substrates, enzymes, and buffers

For enzymatic assays, Kexin (EC 3.4.21.61), Pepsin (EC 3.4.23.1), Subtilisin A (EC 3.4.21.62), Papain (EC 3.4.22.2), and Thermolysin (EC 3.4.24.27) were purchased and used as targets for the potential inhibitory effect of the extracts. Substrates and enzymatic activity conditions for each enzyme are summarized in Table S1.

### Enzymatic activity conditions

Inhibitory activity was assessed by incubating each extract with different peptidases for 5 min in the corresponding buffer at room temperature (see Table S1). Enzymatic activity was measured by adding the corresponding substrate to a final concentration of 1 *K*_*m*_ (Michaelis-Menten constant) and monitoring the product’s appearance over time. Excitation and emission wavelengths used are listed in Table S1.

### Fast protein liquid chromatography

Extracts with desired phenotypic effects were fractionated using Fast Protein Liquid Chromatography (FPLC) with a Superose 6 Increase 10/300 Gl (Cytiva) column, according to the manufacturer’s instructions. Briefly, the column was washed with PBS using two column volumes (CVs) before loading 500 µL (approximately 4 mg of protein) of sample. Fractionating was performed at room temperature using a flow rate of 0.5 mL/min of PBS, and the sample collector was programmed to collect 1 mL per fraction. Progress of the chromatography was monitored using absorbance at 214, 260, 280, and 405 nm.

### Mass spectrometry-based proteomics

Profiling of the mussel extracts was performed as previously described for secretome samples (41). Briefly, 100 µg of crude and clarified mussel extracts were enzymatically digested using a trypsin/Lys-C mixture, followed by desalting and purification using STop And Go Extraction (STAGE)-tips (42). Samples were measured on an Orbitrap Exploris 240 hybrid quadrupole-orbitrap mass spectrometer (Thermo Fisher Scientific) coupled to an Easy-nLC 1200 high-performance liquid chromatography device (Thermo Fisher Scientific). Samples were loaded onto an in-line 75 mm by 50 cm PepMap RSLC EASY-Spray column filled with 2-mm C18 reverse-phase silica beads (Thermo Fisher Scientific). Separated peptides were electro-sprayed into the mass spectrometer with a linear gradient of 3–20% buffer B (80% acetonitrile and 0.5% acetic acid) over a 60-min gradient, followed by a wash with 100% buffer B with a 250 nL/min flow rate. The mass spectrometer was operated in a data-dependent acquisition model and switched between one full scan and MS/MS scans of abundant peaks. Full scans (*m/z* 400 to 2,000) were acquired in the Orbitrap mass analyzer with a resolution of 120,000 at *m/z* 200.

### Mass spectrometry data processing

Analysis of mass spectrometry raw data files was performed using MaxQuant software (version 1.6.0.26) (43). Given that these mussel species do not have complete proteomes annotated, the search was performed using the incorporated Andromeda search engine (44) against the proteins of the *Unionida* group (24,205 sequences; 20 February 2023) for *L. costata* and *D. polymorpha* proteins (265,287 sequences; 20 February 2023) for *D. polymorpha* from UniProt (45) and NCBI (46). The following parameters were included: trypsin enzyme specificity with a maximum of two missed cleavages, a minimum peptide length of seven amino acids, fixed modifications, including carbamidomethylation of cysteine, and variable modifications, including methionine oxidation and N-acetylation of proteins and split by taxonomic ID. Peptide spectral matches were filtered using a target-decoy approach at a false-discovery rate (FDR) of 1%, with a minimum of two peptides required for protein identification. Relative label-free quantification (LFQ) was enabled, and the MaxLFQ algorithm used a minimum ratio count of 1 (47).

### Bioinformatics

Statistical analysis and data visualization of the proteomics data were performed using Perseus (version 2.0.6.0) (48). Data were prepared by filtering for reverse database matches, contaminants, and proteins only identified by site, followed by log_2_ transformation of LFQ intensities. Filtering for valid values (three of four replicates in at least one group) was performed, missing values were imputed from the normal distribution (width, 0.3; downshift, 1.8 standard deviations), and group values were averaged. Significant differences were evaluated by a Student’s *t* test (*P* value ≤ 0.05) with multiple-hypothesis testing correction using the Benjamini-Hochberg (FDR = 0.05 with *S*_0_ = 1) (49). Proteomics profiling was performed in quadruplicate.

### Statistical analysis

For phenotypic assays (i.e., growth, capsule, melanin, and biofilm), data were visualized and statistically analyzed using GraphPad Prism version 9.0 (GraphPad Software, Inc., USA; https://www.graphpad.com/). Statistical tests were performed by one-way analysis of variance (ANOVA) followed by Dunnett’s multiple comparisons tests (treatments against control). *P* values of ≤ 0.05 were considered significant.

## RESULTS

### Protein extracts from mussels affect growth and thermotolerance for *C. neoformans*

Distinct extracts derived from the tissue of mussels were assessed by BCA to determine protein concentrations (Table S2). The results confirm successful protein extraction in crude extracts and as expected, protein loss during clarification. Notably, the highest protein concentration was from crude *D. polymorpha* extracts, and for subsequent experiments, concentrations were normalized across extracts, as appropriate, and dilution series were generated.

**Fig 1:**
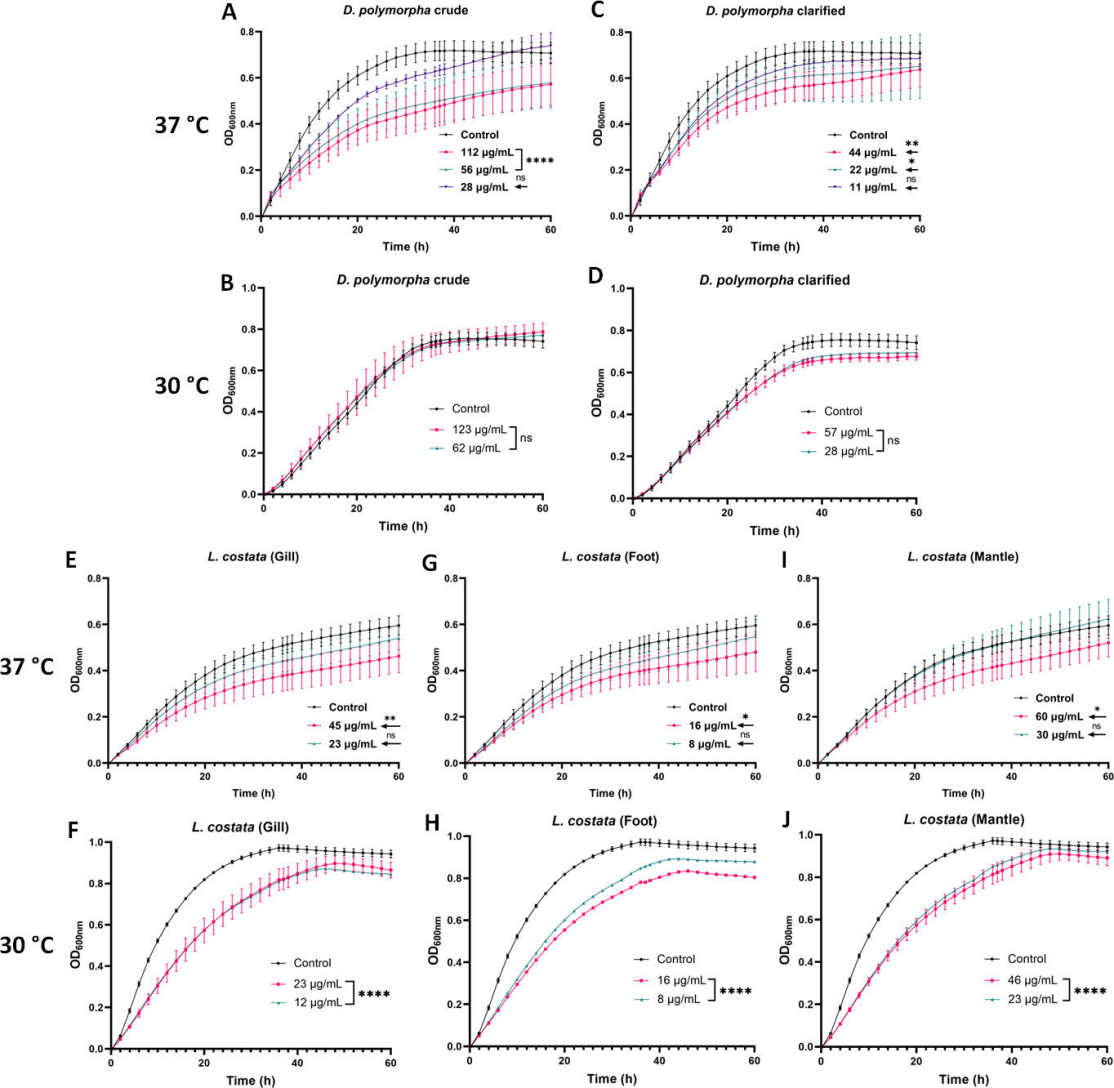
Effect of extracts from mussels on growth (30°C) and thermotolerance (37°C) of *C. neoformans* H99. (A and B) *D. polymorpha* crude; (C and D) *D. polymorpha* clarified; (E and F) *L. costata* (Gill); (G and H) *L. costata* (Foot); and (I and J) *L. costata* (Mantle). Controls consist of *C. neoformans* cells in YNB media without extracts. Experiments were performed in five biological replicates and two technical duplicates. Error bars indicate standard deviation. Statistical analysis was performed using a one-way ANOVA and comparing growth after 24 h and Dunnett’s multiple comparison tests with a *P* value of 0.05; *: *P* < 0.05; **: *P* < 0.01; ***: *P* < 0.001 and ****: *P* < 0.0001. Figures were created using GraphPad Prism 9.

To assess the potential antifungal properties of the mussel extracts on *C. neoformans* growth and tolerance to human temperature (i.e., thermotolerance), we exposed the fungi to different extract concentrations (twofold dilution series from pure extract) at 30°C and 37°C. For the *D. polymorpha* crude extract, we observed inhibition of fungal growth with a significant (*P* < 0.0001) difference in growth using a low extract concentration (56 µg/mL) compared to the control (i.e., no extract present) (Fig. 1A). However, this inhibitory effect was not observed at 30°C (Fig. 1B), supporting a thermotolerance effect. Similar results were observed using the clarified extract of *D. polymorpha* where inhibition was observed above 22 µg/mL at 37°C (Fig. 1C), but no growth defect was observed at 30°C (Fig. 1D). On the contrary, for *L. costata*, we observed extracts with significant (*P* < 0.05) inhibition using low protein concentration ranges (8–60 µg/mL) of each dissected tissue at 30°C and to a lesser extent at 37°C (Fig. 1E through J), suggesting a role in fungal growth and thermotolerance. Interestingly, we observed a dose-dependent reduction in fungal growth under the tested conditions.

### Protein extracts from *D. polymorpha* affect *C. neoformans* capsule but not melanin production

**Fig 2:**
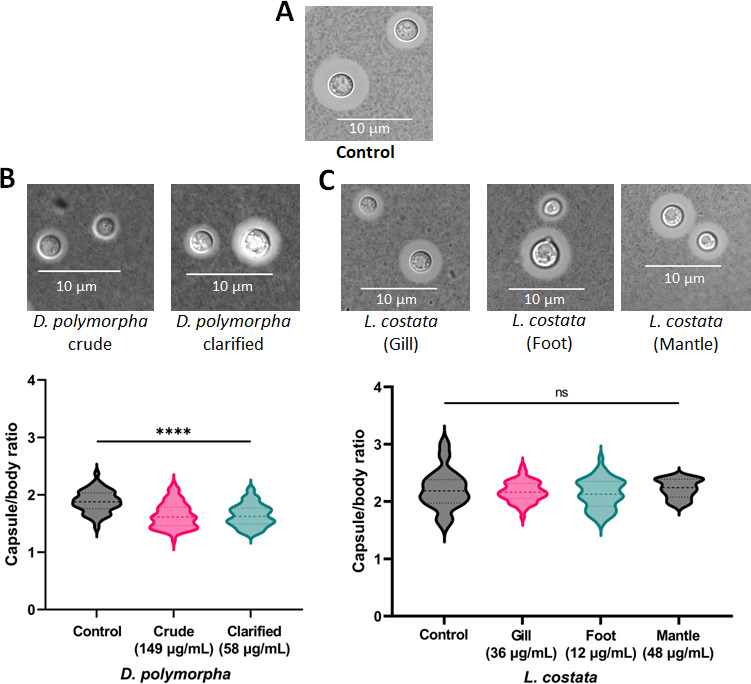
Effect of mussel extracts on polysaccharide capsule production in *C. neoformans* at 37°C. (A) Control (No treatment). (B) Effect of crude and clarified extracts from *D. polymorpha*. (C) Effect of crude extracts from different morphological regions of *L. costata*. The cells were visualized using India ink (1:1 ratio) and a DIC microscope with an oil 63× objective. Error bars indicate standard deviation. Statistical analysis was performed using a one-way ANOVA and Dunnett’s multiple comparison tests with a *P* value of 0.05. **: *P* < 0.01; ***: *P* < 0.001 and ****: *P* < 0.0001. Approx. 50–60 cells were measured per treatment, with a ratio of total cells (including capsule) to cell body presented. Experiments were performed in biological triplicate and two technical duplicates. The figures were created using GraphPad Prism 9.

To assess the impact of mussel extracts on another critical virulence determinant for *C. neoformans*, we explored the inhibition of polysaccharide capsule production. We observed that both crude and clarified extracts from *D. polymorpha* significantly (*P* < 0.0001) inhibited the production of polysaccharide capsules by approx. 26% compared to the control (Fig. 2A and B). However, in the presence of *L. costata* extracts, we did not observe a significant reduction in capsule size across the treatments (Fig. 2C). In this assay, we used *C. neoformans* H99 cultured in LIM as the control for comparison to extract treatments. Notably, none of the extracts altered melanin production (Fig. S1). These results highlight a prominent effect of the extracts against the polysaccharide capsule of *C. neoformans* using different mussel species, which supports an opportunity to weaken fungal virulence determinant production within the host and promote fungal clearance.

### Mussel extracts impact cryptococcal biofilm formation but not disruption in pre-formed communities

**Fig 3:**
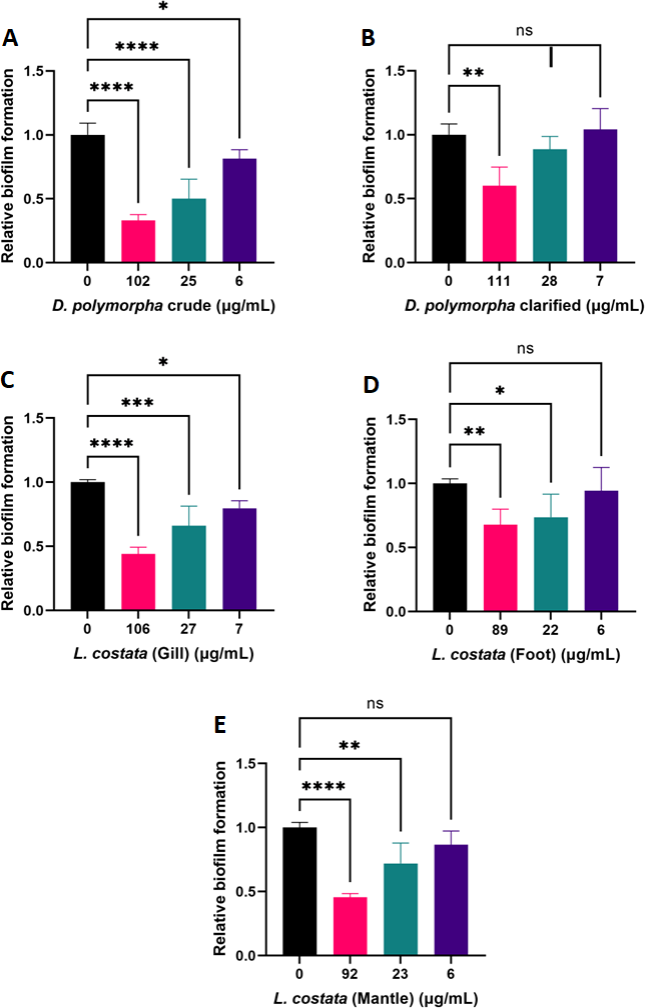
Effect of mussel extracts on biofilm formation of *C. neoformans* H99 at 37°C. (A) *D. polymorpha* crude; (B) *D. polymorpha* clarified; (C) Crude *L. costata* (Gill); (D) Crude *L. costata* (Foot); and (E) Crude *L. costata* (Mantle). Biofilm formation on each condition was normalized to the control without any extract (0 µg/mL). Experiments were performed in biological quadruplicate and technical duplicate. Error bars indicate standard deviation. Statistical analysis was performed using a one-way ANOVA and Dunnett’s multiple comparison tests with a *P* value of 0.05. **: *P* < 0.01; ***: *P* < 0.001 and ****: *P* < 0.0001. Figures were created using GraphPad Prism 9.

To evaluate the potential effects of mussel extracts on cryptococcal biofilm formation, the cells were incubated with extracts from *D. polymorpha* and *L. costata* at 37°C for a 48-h period. Here, we detected a significant reduction of biofilms after treatment with crude and clarified extracts of *D. polymorpha,* respectively (Fig. 3A and B). Similarly, we also observed a significant reduction in biofilm biomass upon treatment with segmented regions of *L. costata* (Fig. 3C through E). We acknowledge that differences in the inhibition of biofilm formation may be related to the inhibition of fungal growth; however, the lower extract concentrations tested in the biofilm assay (that showed inhibition, which was not observed in the growth assays at higher extract concentrations) support distinct mechanisms of action of the extracts between the two assays. Next, given our observations of reduced biofilm biomass upon extract treatment, we further assessed if extracts eliciting a dose-dependent inhibitory effect on biofilm formation could also disrupt pre-formed fungal biofilms at 37°C. We did not observe a significant effect on pre-established biofilms upon extract treatment (Fig. S2).

### Resistance to membrane stress is influenced by extract treatment

To identify mechanisms that may explain the inhibitory effects of each extract, *C. neoformans* cells were briefly treated using sub-inhibitory concentrations of the extracts and exposed to osmotic and membrane stressors (Fig. S3A). This evaluation revealed that extracts from the foot and mantle of *L. costata* affect the pathogen’s ability to resist membrane stress caused by the chaotropic agent, SDS (0.01%) (Fig. S3B). These effects were comparable to 8 µg/mL (1 minimal inhibitory concentration [MIC]_90_) of fluconazole, a known membrane disruptor. However, when exposed to osmotic stress (1 M NaCl), extracts did not produce a visible effect compared to 10 µg/mL of Amphotericin B (5 × MIC_90_), an osmotic disrupter (Fig. S3C) (50). Finally, we assessed the extracts' effect on secretory mechanisms by detecting urease activity. We did not observe a notable effect in this assay compared to the urease inhibitor, EDTA (5 mM) (Fig. S3D).

### Mussel extracts reduce the survival of *C. neoformans* H99 upon co-culture with macrophages

**Fig 4:**
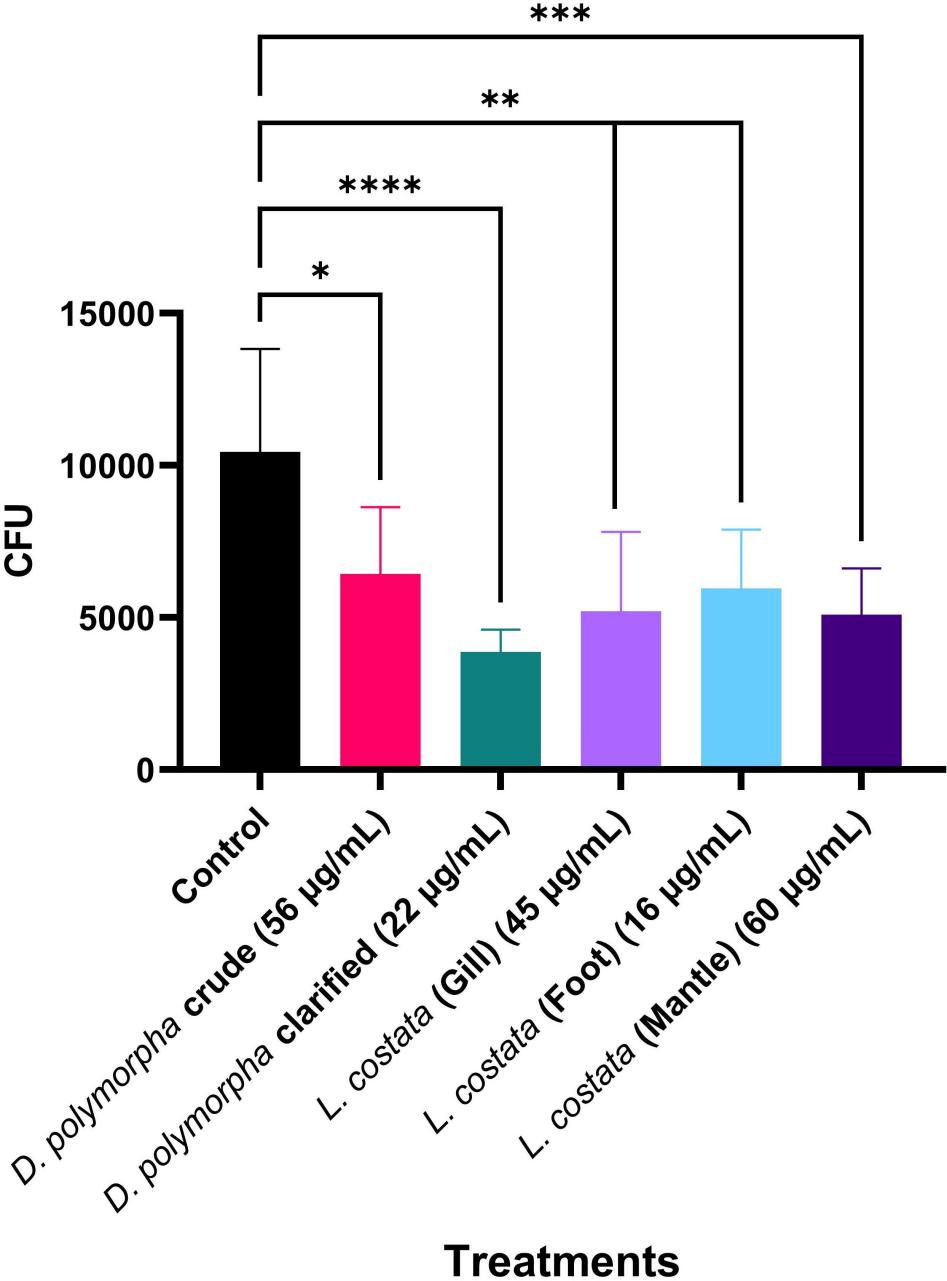
Effect of mussel extracts on *C. neoformans* H99 survival after co-culture with BALB/c macrophages. Experiments were performed using three biological replicates and technical duplicates. Control indicates natural death after the incubation period without extract treatment. Error bars indicate standard deviation. Statistical analysis was performed using a one-way ANOVA and Dunnett’s multiple comparison tests with a *P* value of 0.05; *: *P* < 0.05; **: *P* < 0.01; ***: *P* < 0.001; and ****: *P* < 0.0001. Figures were created using GraphPad Prism 9.

Given the implication of the extracts on significantly reducing fungal growth, thermotolerance, and capsule production, we hypothesize that treatment increases fungal susceptibility to host defenses. Thus, we evaluated the effect of extract treatment on fungal survival within macrophages (i.e., a first line of defense within the mammalian system to clear pathogens) (51). Since *C. neoformans* survives within macrophages and uses the cells as a strategy to invade the central nervous system, we assessed the ability of the mussel extracts to reduce the fungal burden within an *in vitro* macrophage assay (52). In this assay, cryptococcal cells were co-cultured with immortalized macrophages from BALB/c mice before incubation with mussel extracts, followed by quantification of the infection burden using CFU counts (Fig. 4). Crude extracts from *D. polymorpha* had a significant (*P* < 0.05) inhibitory effect on the survival of *C. neoformans*. This inhibitory effect was enhanced after clarification. Furthermore, all extracts from *L. costata* significantly reduced the survival of cryptococcal cells co-cultured with macrophages. We also assessed the possible cytotoxic effects of the extracts on mammalian cells (i.e., BALB/c macrophages) by quantifying LDH release. Notably, we did not observe significant differences in LDH release compared to the untreated control for all extracts except the *L. costata* gill (Fig. S4).

### Protein extracts from mussels increase the susceptibility of *C. neoformans* to fluconazole treatment in the presence of macrophages

**Fig 5:**
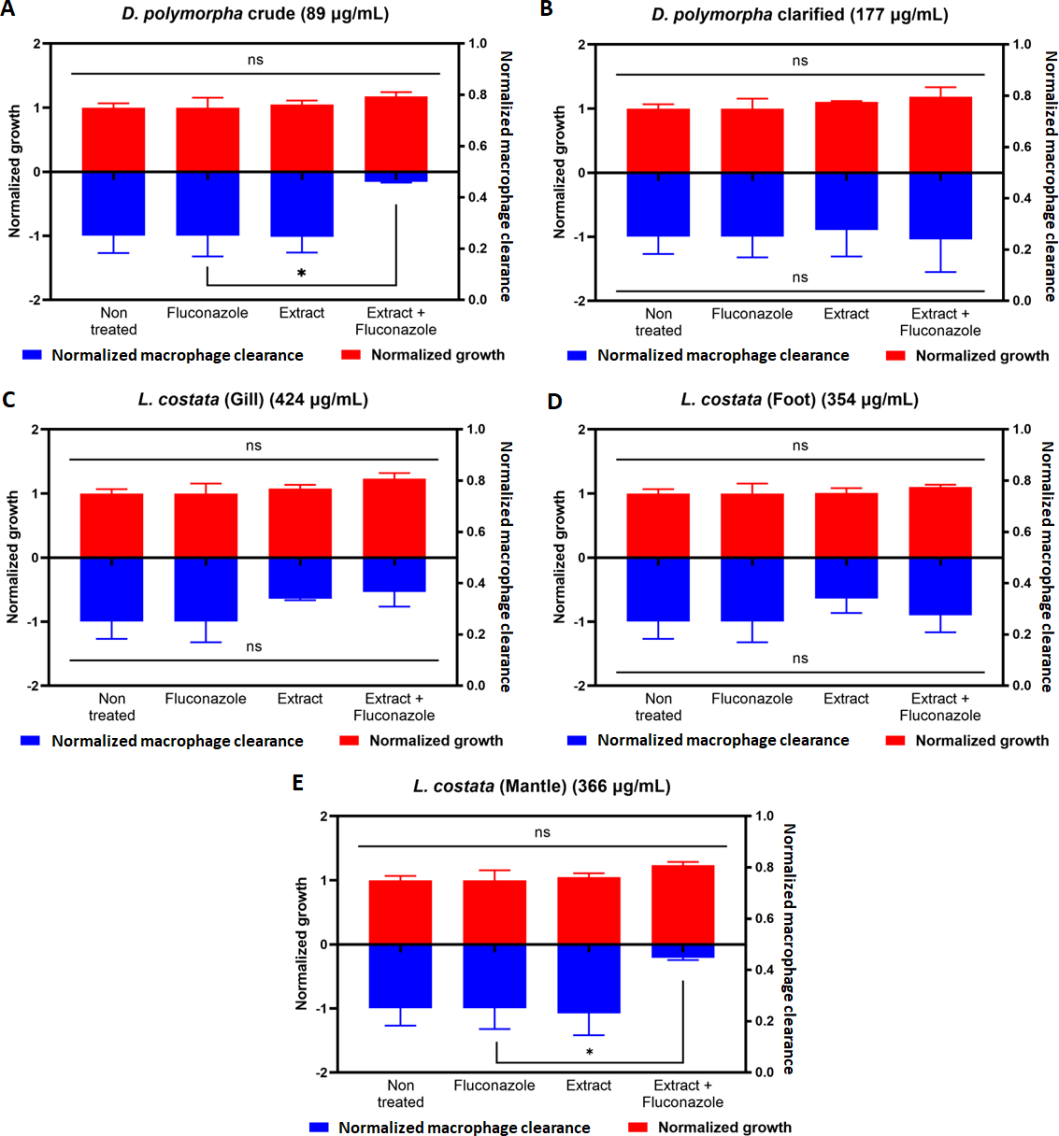
Effect of mussel extracts on fluconazole susceptibility and macrophage clearance of the fluconazole-resistant CL strain. (A) *D. polymorpha* crude; (B) *D. polymorpha* clarified; (C) Crude *L. costata* (Gill); (D) Crude *L. costata* (Foot); and (E) Crude *L. costata* (Mantle). Growth at 37°C was monitored using OD_600nm_. Survival after macrophage infection was monitored by quantification of CFU after 15 h of infection. Each treatment with extract was normalized with the non-treated sample, and extract combined with fluconazole (64 µg/mL) was normalized using a fluconazole-only sample (64 µg/mL). Statistical analysis was performed using a Kruskal-Wallis test and Šídák multiple comparison test with a *P* value of 0.05; *: *P* < 0.05; **: *P* < 0.01; ***: *P* < 0.001; and ****: *P* < 0.0001. Figures were created using GraphPad Prism 9.

Given our observations of disruption to cell membrane integrity using external stressors, we next aimed to assess the potential of mussel extracts to influence susceptibility to fluconazole in resistant *C. neoformans* strains. Fluconazole is a mainstream antifungal that inhibits Erg11, a protein involved in ergosterol synthesis, to cause increased membrane instability and delayed development (53). Here, we monitored the growth of the fluconazole-resistant CL *C. neoformans* H99 strain treated with each extract, fluconazole (64 µg/mL), or a combination of extract and fluconazole. We did not observe a significant difference in the susceptibility of the CL strain to fluconazole in the presence of the extracts (Fig. 5A through E).

Next, given our observation of macrophage clearance on fungal strains following extract treatment, and the connection between fluconazole susceptibility and the immune system (54, 55), we evaluated the potential for interactions between fluconazole and macrophages to influence fungal survival. In this assay, fungal cells were co-cultured with macrophage followed by treatment with extracts, fluconazole (64 µg/mL), or the combination of extracts and fluconazole. Macrophages were lysed and CFU counts of *C. neoformans* were performed to assess the potential for macrophages to influence the fluconazole susceptibility of the CL strain. We observed a significant reduction in fungal cell survival when extract treatment was combined with fluconazole in the presence of macrophages for the crude extracts of *D. polymorpha* (Fig. 5A). However, we did not observe a significant effect using clarified extracts of *D. polymorpha* (Fig. 5B) or using extracts from the gill and foot of *L. costata* (Fig. 5C and D). Extracts from the mantle of *L. costata* also increased macrophage clearance upon fluconazole treatment (Fig. 5E). These data support a role for selected extracts in promoting fluconazole susceptibility in the presence of macrophages.

### Mussel extracts inhibit distinct commercial orthologs of virulence-related peptidases in *C. neoformans* to confirm phenotypic assessments

To uncover potential molecular mechanisms driving the phenotypic effects of the mussel extracts, we evaluated their inhibitory activity against commercial orthologs of virulence-related peptidases (14). For instance, we assessed metallopeptidase activity (essential for fungal brain invasion) and aspartic proteolytic activity (involved in fungal escape from macrophages) (15, 56, 57). Notably, previous research reported that inhibition of these enzymes using natural compounds reduced brain burden and virulence in a mouse model (58). We measured the IC_50_ values of each extract against commercial enzymes (commonly derived from *S. cerevisiae*) from different catalytic mechanism classes [i.e., Pepsin (EC 3.4.23.1), Subtilisin A (EC 3.4.21.62), Papain (EC 3.4.22.2), and Thermolysin (EC 3.4.24.27)] (Table 1). Notably, although the enzymes tested are not derived from *C. neoformans*, the enzymes constitute family representatives related to our study (59). Here, we detected a high inhibitory activity of *L. costata* extracts against Subtilisin-like enzymes (associated with fungal quorum sensing) (60). We also measured a notable reduction in the proteolytic activity of the Papain-like enzyme (associated with capsule production) using clarified extracts of *D. polymorpha* (61). Similarly, we observed that clarified *D. polymorpha* extracts and *L. costata* (mantle) have moderate inhibitory activity against Pepsin-like enzymes (associated with biofilm formation and macrophage resistance) (15). Finally, we detected that clarified extracts from *D. polymorpha* and extracts from *L. costata* have inhibitory activity against Thermolysin-like peptidases (related to fluconazole resistance) (62). Assays with no change in fungal phenotypic profiling were observed for the respective extract and preparation; activity was not assessed (i.e., “ND”). If the intrinsic proteolytic activity was detected, IC_50_ values could not be measured and were defined as “IPA.” Taken together, these findings connect four inhibitory activity profiles of the mussel extracts with the phenotypic observations associated with changes in fungal virulence determinant production.

**TABLE 1:** IC_50_ values of mussel extracts toward commercial orthologs of virulence-related peptidases defined in *Cryptococcus neoformans^a^*

Mussel extracts	Subtilisin-like (quorum sensing)	Papain-like (capsule)	Pepsin-like (biofilm)	Thermolysin-like (drug resistance)
*D. polymorpha* crude	ND	IPA	ND	ND
*D. polymorpha* clarified	ND	4.07 µg/mL	80 µg/mL	146 µg/mL
*L. costata* (gill)	3.39 µg/mL	ND	ND	74.1 µg/mL
*L. costata* (mantle)	2.26 µg/mL	ND	48 µg/mL	60.5 µg/mL
*L. costata* (foot)	8.84 µg/mL	ND	ND	26.6 µg/mL

^*a*^ IPA: intrinsic proteolytic activity, ND: not assessed.

### Proteomic profiling defines signatures supporting phenotypic fungal modulation by mussel extracts

**Fig 6:**
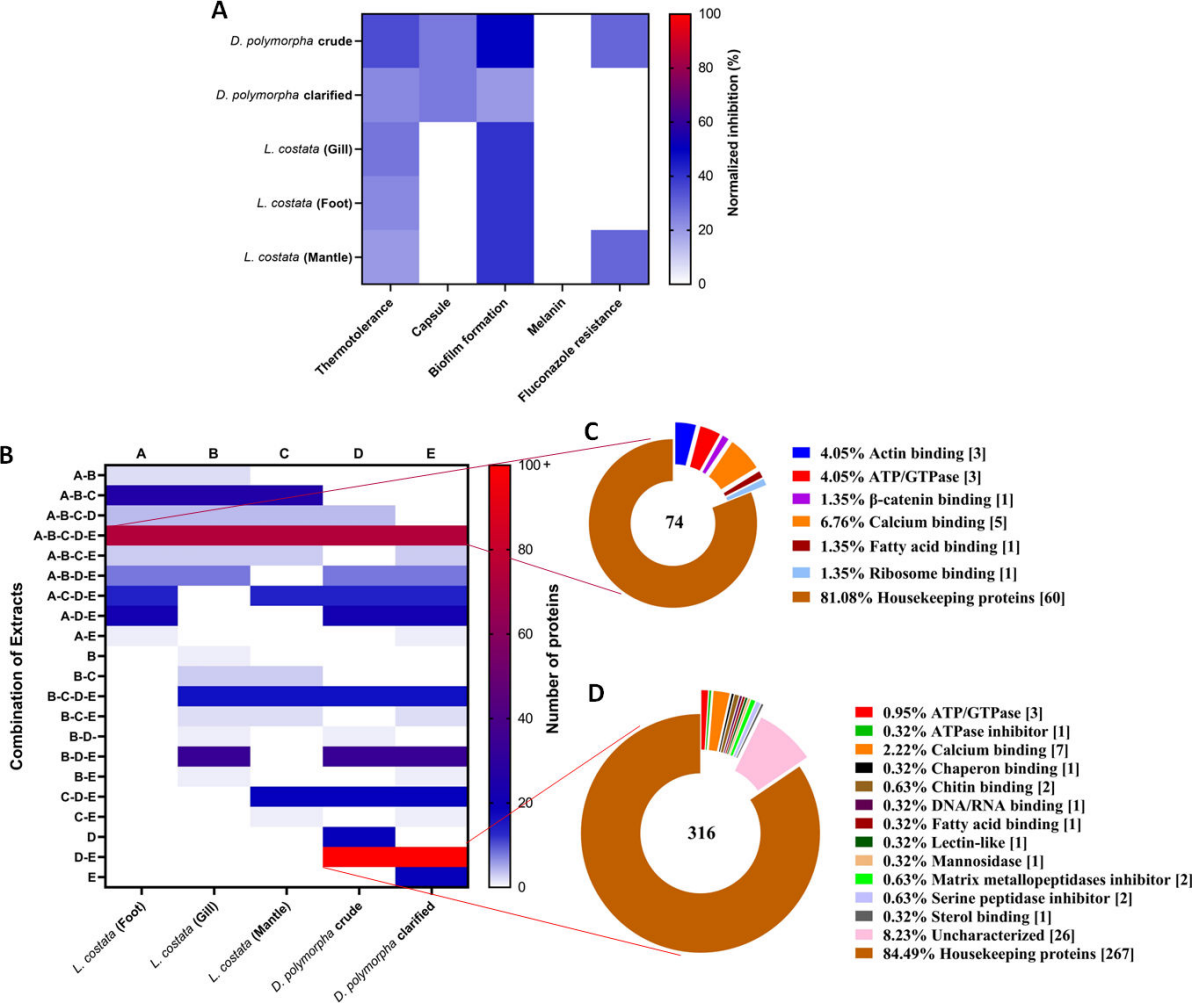
Correlation of phenotypic extract effects on *C. neoformans* by defining proteomic signatures. (A) Summary of the inhibitory effect of protein extracts on *C. neoformans* virulence factors. Values were normalized to the respective control. (B) Number of proteins shared among mussel extract combinations. (C and D) Pie charts show the distribution of proteins identified in the mussel extracts, i.e., *D. polymorpha* and *L. costata*, based on Gene Ontology Molecular Function (GOMF) and protein names. Proteomics data were analyzed with MaxQuant. Experiments were performed in biological quadruplicate. The figures were created using GraphPad Prism 9.

Given our phenotypic findings across experimental assays, we integrated inhibition profiles across phenotypic data sets normalized to untreated *C. neoformans* H99 (Fig. 6A). These data reported that for thermotolerance, all mussel extracts had an impact on *C. neoformans* cells grown at 37°C. For capsule production, the crude and clarified extracts from *D. polymorpha* significantly reduced capsule production, while none of the extracts altered melanin production. Additionally, we observed that all extracts impacted biofilm formation without a disruptive effect. Moreover, susceptibility to fluconazole in the *C. neoformans* CL strain was influenced by the crude extract of *D. polymorpha* and the crude extract of *L. costata*’s mantle within macrophage.

Next, bottom-up mass spectrometry-based proteomics profiling was performed to identify potential candidate proteins present in the mussel extracts, specifically, proteins driving the phenotypic, virulence, and resistance mechanisms evaluated above. This analysis revealed over 500 proteins across the two mussel species detectable within the extracts. Based on these results, we defined each of the mussel extracts (i.e., *D. polymorpha* and *L. costata*) and the respective preparation (i.e., crude and clarified) or morphological region (i.e., gill, foot, or mantle) from (A–E) (Table S3). We identified proteomics signatures for each combination (Fig. 6B). In this context, unique and common proteins (0 to over 100) were determined for each phenotype-associated classification.

To uncover the identities of these candidate proteins and explore their potential relationship with phenotypic changes in *C. neoformans*, we used Gene Ontology Molecular Function (GOMF) affiliations and defined protein names. For instance, all extracts with inhibitory effects on thermotolerance and biofilm formation shared 74 unique proteins with functions related to actin binding (approx. 4%), β-catenin binding (approx. 1.3%), and calcium binding (approx. 6.7%) (Fig. 6C). Likewise, extracts from crude and clarified *D. polymorpha* with effects on capsule production shared 316 proteins involved in multiple molecular processes, such as chitin-binding (approx. 0.6%), mannosidase activity (approx. 0.3%), peptidase inhibitors (approx. 1.2%), and calcium binding (approx. 2.2%) (Fig. 6D). Proteins with no notable implication in these antifungal effects were grouped as “housekeeping proteins” for the purpose of this study. Taken together, these findings define core and unique proteome signatures across the extracts with putative roles driving the phenotypic observations against cryptococcal virulence determinants.

### Extract fraction profiling provided mechanistic insight into inhibitory effects on capsule production

**Fig 7:**
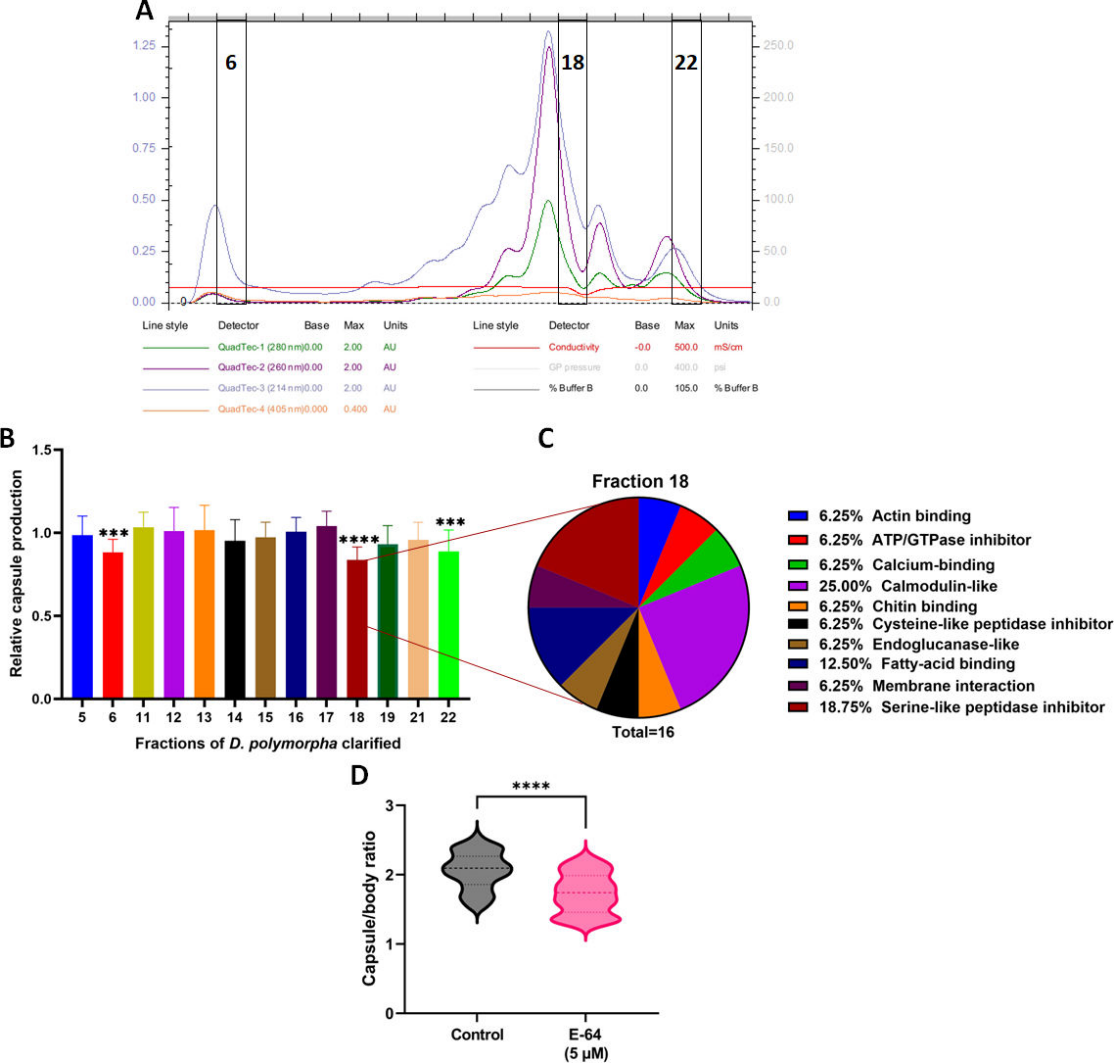
Identification of candidates from the clarified extract of *D. polymorpha* with effects on capsule production in *C. neoformans* H99. (A) Chromatogram of the size exclusion chromatography using clarified extracts of *D. polymorpha*. (B) Effect of fractions in capsule production normalized and compared to a non-treated sample. (C) Pie charts show the distribution of proteins identified in fraction 18 based on Gene Ontology Molecular Function (GOMF). Proteomics data were analyzed with MaxQuant. Experiments were performed in a biological quadruplicate. (D): Effect of E-64 in the capsule production in *C. neoformans*. Error bars indicate standard deviation. Statistical analysis was performed using a one-way ANOVA and Dunnett’s multiple comparison tests with a *P* value of 0.05. ****: *P* < 0.0001. Approx. 50–60 cells were measured per treatment. Experiments were performed in biological triplicate and technical duplicate. The figures were created using GraphPad Prism 9.

Based on the significant inhibitory effects observed in capsule production using the clarified extracts of *D. polymorpha,* we further fractionated this extract using FPLC (Fig. 7A). To identify the active fraction behind these effects, we reassessed the inhibitory activity of the resulting fractions against the capsule production using *C. neoformans* H99 WT (Fig. 7B). Here, we observed that fractions 6, 18, and 22 had significant inhibitory effects on capsule production, with fraction 18 displaying the most considerable inhibition. Furthermore, we performed proteomic profiling on fraction 18 to identify potential active molecules (Fig. 7C). This profiling revealed the presence of 243 proteins that were prioritized by molecular weight (5–50 kDa), sequence coverage (higher than 10%), and MS-MS count (at least 2) to 115 proteins. From these 115 candidates, 15 were selected as potential active molecules based on assigned GOMF. Here, we detected four calmodulin-like proteins (25%), one endoglucanase (6.25%), and one cysteine-like peptidase inhibitor (6.25%) among other proteins (Table S4).

Finally, given that *C. neoformans* capsule production is associated with a cysteine-like peptidase, CNAG_05601 (35), we selected the identified cysteine-like peptidase inhibitor from fraction 18 as a proof-of-concept to validate our findings. Here, we assessed the effect of purified E-64, a general cysteine peptidase inhibitor, against *C. neoformans* capsule production. We observed a significant reduction in capsule to cell size ratio upon treatment (Fig. 7D).

## DISCUSSION

Mussels filter bacteria, protozoans, algae, and other organic matter out of the water with food particles carried to the mouth by tiny hairlike cilia located on the gills (63). Material not taken into the digestive tract is released in the mantle cavity to be periodically expelled through the inhalant aperture (64, 65). To deal with potential pathogens, invertebrates, such as mussels, do not possess an adaptative immune system but instead rely on a potent innate immune system and a vast array of antibiotic compounds selectively adapted for defense without adversely affecting the host cells (66–68). These inherent protective strategies of invertebrates present a unique opportunity to leverage the intelligent design of natural sources and explore putative antimicrobial properties. In this study, we investigated the putative therapeutic properties of compounds derived from mussels toward inhibition of virulence factors produced by the human pathogen *C. neoformans*, and we defined and validated core and unique proteome signatures that drive such beneficial properties. We identified and confirmed the role of a cysteine-like peptidase inhibitor in the reduction of *C. neoformans* capsule production. We acknowledge that while additional low molecular weight molecules may also be implicated in these findings, they were not the focus of this investigation but present ample research opportunities in the future.

### Inhibition of growth and thermotolerance

One of the first lines of defense employed by humans against pathogens is a relatively high internal temperature. To succeed, human pathogens, including *C. neoformans,* have developed thermotolerance mechanisms to survive in this environment and advance through infection. An important temperature-sensing signaling cascade includes the action of calcineurins, a Ca^2+^-dependent serine/threonine phosphatase, required not only for growth inside the host but also for virulence and mating within the environment (69). In this paper, we observed a significant inhibition of fungal growth at 37°C following treatment with each extract. Furthermore, extracts of *D. polymorpha* did not have a significant effect on the growth of *C. neoformans* at 30°C, indicating the presence of molecules with specific effects on thermotolerance mechanisms rather than fungal growth. On the other hand, extracts from *L. costata* had similar effects at both temperatures, suggesting putative inhibitory mechanisms on general growth-related processes. We identified five calmodulin-like proteins commonly produced by the extracts using bottom-up mass spectrometry-based proteomics. These proteins may compete for intracellular Ca^2+^ and, thus, impair the thermotolerance role of calcineurin in *C. neoformans*. Inhibition of these enzymes has been widely employed by drugs like cyclosporin, a natural compound used to immunosuppress and treat multiple human diseases (70, 71). Very recently, cyclosporin was found to have various effects on virulence and cell morphology in *C. neoformans,* including the cell wall and the polysaccharide capsule (11).

### Impact on polysaccharide capsule production

Cryptococcal capsule, with roles in virulence, immune system evasion, and low resemblance to extracellular macromolecules in mammalian cells, constitutes one of the most attractive anti-virulence targets of this pathogen (9). The polysaccharide capsule is in the outer layer of the extracellular region of *C. neoformans*, and is attached to the cell wall, making it susceptible to changes in the cell wall composition or morphology (54, 55). In this study, we observed a significant reduction in capsule production using extracts of *D. polymorpha* but not *L. costata*. Proteomic analysis revealed 316 unique proteins in *D. polymorpha* samples with potential implications in the capsule inhibitory effect. For instance, a chitin deacetylase was identified, which converts chitin into chitosan, affecting the morphology of this polymer (72). Although this effect was not enough to impair *C. neoformans* ability to tolerate osmotic stress, it may impact cell wall morphology, which is essential for capsule formation (73, 74). Similarly, calcineurins are calcium-binding proteins involved in capsule production, and their inhibition is known to cause cell wall impairment (11). Here, we also observed seven calcium-binding proteins that may impact the role of calcineurin and, eventually, capsule production (8).

Using enzymatic approaches, we also detected the presence of strong papain-like (C1 cysteine-type) proteolytic activity inhibition in clarified extracts of *D. polymorpha*. Notably, Rim13 (CNAG_05601), an intracellular cysteine peptidase, participates in the activation of Rim101, a transcriptional factor with multiple functions, including the formation of the cell wall and, thus, polysaccharide capsule (35). During a previous study, we also detected cysteine protease inhibitors from invertebrates with potential roles in capsule inhibition in *C. neoformans* (29). The present study confirmed a connection between cysteine peptidase inhibition and capsule reduction in *C. neoformans*. Another potential mechanism of capsule inhibition identified through our fraction profiling was an endoglucanase-like enzyme from clarified extracts of *D. polymorpha*. Endoglucanases are low-specificity enzymes that digest large polysaccharides and may affect the stability of the cell wall (composed primarily by β−1,6-glucans) in *C. neoformans* and, thus, the capsule attachment (75, 76). Future studies may focus on fungicidal effects using endoglucanases, as previously reported (77). Notably, we could not measure the inhibitory activity of crude extracts from *D. polymorpha* against papain-like enzymes due to intrinsic proteolytic activity. Conversely, the absence of papain-like enzymes in the clarified extract highlights the success of our attempt to discard high molecular weight proteins (e.g., peptidases) through thermal clarification.

### Inhibition of biofilm formation

One of the evasion mechanisms used by bacterial and fungal human pathogens to avoid the immune system is the formation of biofilms (78). This highly compacted structure also increases antibiotic resistance through a reduction in drug access to the intracellular space, which supports the evolution of cells able to withstand antimicrobial treatment (17). To form a dense biofilm community, *C. neoformans* produces and secretes multiple proteins (e.g., aminotransferases and aspartic proteases) and carbohydrates (e.g., glucose, xylose, and mannose) to develop a complex extracellular matrix that promotes adhesion (79, 80). In this study, we observed variable inhibitory effects on biofilm formation by *C. neoformans* across the mussel extracts. We acknowledge the limitations of our approach, including that inhibition of biofilm formation may be associated with inhibition of fungal growth. However, for the crude *C. polymorpha* extract, we observed a significant reduction in biofilm formation at the lowest tested concentration (i.e., 6 µg/mL), whereas we did not observe a significant reduction in fungal growth (i.e., 28 µg/mL). We observed similar patterns of inhibition for *L. costata* gill and mantle. These findings suggest that factors aside from the inhibition of fungal growth are influencing inhibition of biofilm formation upon extract treatment. Our proteomics profiling identified β-catenin binding proteins, which are involved in cadherin interaction with the cytoskeleton and cell-cell adhesion mechanisms in animal cells (81). In yeasts, such as *Candida albicans,* cadherin-like proteins (i.e., agglutinin-like proteins) are essential for biofilm formation (82). The presence of β-catenin binding in these mussels may impact biofilm formation by inhibiting cadherin-like proteins and cell-cell interaction mechanisms.

As stated above, biofilms are condensed structures that diminish the efficacy of antifungal drugs, turning their disruption into a desirable but hard-to-find activity in new treatments (17). In this context, aspartic peptidases are important for establishing fungal biofilms and inhibiting such enzymes can make biofilms more susceptible to antifungal agents in *C. albicans* (20). Notably, *C. neoformans* forms biofilms and secretes an aspartic peptidase (CNAG_5872) that is crucial to its survival inside macrophages to escape the immune action (83). Here, we detected the presence of pepsin-like (A1 aspartic type) peptidase inhibitors in the extracts that could explain the effects on biofilm and macrophage clearance. Aspartic inhibitors could be a crucial discovery not only to modulate cryptococcal biofilms but also to treat the deadly combination with HIV, an aspect that has been explored by other researchers but encourages further study (83, 84).

### Reduction of macrophage burden

Once *C. neoformans* enters the lungs, the fungus faces primary lines of defense, including alveolar macrophages; however, *C. neoformans* can resist macrophage immune actions (52), such as high acidic conditions inside the phagolysosome (85). In this context, *C. neoformans* can form biofilms, produce polysaccharide capsule, and secrete acid peptidases (83, 86) as protective mechanisms. Here, we observed that all mussel extracts significantly reduced the survival of *C. neoformans* inside these immune cells; only extracts from the gill of *L. costata* produced a significant increase in macrophage cell death compared to the control. Although the difference in cell death was statistically significant, the increment was not high (from 4% to 6%). Using the same concentration, extracts from the gill of *L. costata* killed 2% of viable macrophage cells but 50% of cryptococcal cells. This suggests this extract possesses an approximate therapeutic index (TI) (ratio between the dose required for efficacy vs. toxicology) of 25. While there is no defined threshold for selection of new drugs, and it is a complex parameter with multiple factors to consider, the difference in TI and lack of new antifungal treatments against *C. neoformans* stress the importance of further characterization of these extracts (87).

Given these *in vitro* phenotypic observations, crude extracts from *D. polymorpha* likely reduced fungal burden through inhibitory effects on thermotolerance and capsule production. On the other hand, clarified extracts from *D. polymorpha*, with an enhanced burden reduction, affected cryptococcal survival by impairing biofilm formation and the inhibition of extracellular aspartic peptidases, such as May1 (CNAG_05872). Similarly, extracts from *L. costata* affected cryptococcal survival inside macrophages. The capacity of these mussel extracts to disrupt *C. neoformans* defenses against the immune system highlights *D. polymorpha* and *L. costata* as promising sources of antifungal compounds to use as a treatment for cryptococcal and similar fungal infections.

### Effects on fluconazole susceptibility

With more than $1B US in annual sales, fluconazole is one of the most prescribed drugs against human fungal pathogens, including *C. neoformans* and *C. albicans* (88, 89). However, the emergence of resistant strains with higher tolerance to this drug is a serious threat to current treatments, especially in less-developed countries. Our proteomics profiling detected four calcium-binding proteins that may impair secretory mechanisms used by *C. neoformans* to form extracellular structures, such as the capsule or the cell wall, and potentially impacting drug resistance (11, 90). Another possible mechanism may be membrane destabilization by extracts from the *L. costata* mantle, which can also affect the permeabilization of the cell wall and capsule (90).

Interestingly, we observed a significant reduction of macrophage burden in the fluconazole-resistant CL strain when crude extracts of *D. polymorpha* and the mantle of *L. costata* were combined with fluconazole. However, we did not observe a similar pattern when these two extracts acted on the fungal cells alone. These data suggest that resistance mechanisms to fluconazole are influenced by the presence of macrophages where complex mechanisms of fungal clearance occur (91). Among the significantly abundant proteins in the crude extracts of *D. polymorpha*, we observed two chitin deacetylases and one endoglucanase that may affect the integrity of both the cell wall and the capsule, increasing accessibility of the fungus by fluconazole and, thus, enhancing the clearance mechanisms employed by macrophages (e.g., oxidative stress and defensins) (91). Similarly, extracts from the mantle of *L. costata* possess a unique Kazal-type serine peptidase inhibitor compared to the gill and foot. This inhibitor may affect the activity of a cell-associated serine peptidase, Pqp1, which is involved in quorum sensing mechanisms and formation of extracellular structures, such as cell wall and capsule, in *C. neoformans* (92).

### Conclusion

With the emergence of antifungal-resistant strains, targeting virulence determinants represents a novel approach that could disarm human fungal pathogens, such as *C. neoformans*, with less selective pressure towards resistance. Mussels, like other invertebrates, represent promising sources of antifungal compounds against *C. neoformans*. In this study, we differentially extracted aqueous soluble compounds from two freshwater mussels, *D. polymorpha* and *L. costata*, and explored their potential anticryptococcal properties. Extracts from both mussels have significant inhibitory activity against the thermotolerance and biofilm formation in *C. neoformans*, possibly through inhibition of calcineurin-like enzymes or extracellular peptidases. Likewise, extracts from *D. polymorpha* impaired the formation of the polysaccharide capsule potentially through inhibition of cysteine peptidases involved in the Rim pathway. Furthermore, extracts from both mussel species impacted fluconazole susceptibility in a clinical strain exposed to macrophages, supporting increased susceptibility to fluconazole in combination with extracts and macrophages. By further understanding the mechanisms driving the antifungal activity of mussels, we may support the discovery and development of novel anti-virulence treatments against fungal disease.

## Data Availability

The RAW and affiliated files were deposited into the publicly available PRIDE partner database for the ProteomeXchange consortium with the data set identifier: PXD043643.

## SUPPLEMENTAL MATERIAL

Supplemental tables and figures spectrum.02841-23-s0001.docx Tables S1-S3; Figures S1-S4.

Table S4 spectrum.02841-23-s0002.xlsx Proteins identified in fraction 18.

ASM does not own the copyrights to Supplemental Material that may be linked to, or accessed through, an article. The authors have granted ASM a non-exclusive, world-wide license to publish the Supplemental Material files. Please contact the corresponding author directly for reuse.
