# Supplementary material for: Natural compounds from freshwater mussels disrupt fungal virulence determinants and influence fluconazole susceptibility in the presence of macrophages in Cryptococcus neoformans
Source: Microbiol Spectr. 2024 Feb 8;12(3):e02841-23. doi: 10.1128/spectrum.02841-23 (PMC10913472; doi:10.1128/spectrum.02841-23)
Supplement: Supplemental tables and figures — Tables S1-S3; Figures S1-S4. [file spectrum.02841-23-s0001.docx]

# Supplemental tables and figures

**Table S1: Conditions for enzymatic activity assay**

| **Enzyme** | **Substrate** | **Km**  **(mM)** | **Buffer and Temperature** | **Excitation/ Emission** | **Reference** |
| --- | --- | --- | --- | --- | --- |
| Pepsin from porcine (Sigma) | MOCAc-APAKFFRLK-(Dnp)-NH (BioSource) | 6.17x10^-3^ | 50 mM sodium citrate pH:3,  37 ˚C | 328/393 | (1) |
| Subtilisin A  (Sigma) | Suc-AAPF-pNA (Sigma) | 0.2 | Activity: 100 mM Tris-HCl pH:8.6, 25 ˚C | 405 nm (chromogenic) | (2) |
| Papain  (Sigma) | Z-FR-AMC  (MD Systems) | 0.06 | 100 mM sodium phosphate buffer, pH 6.5, 2 mM DTT, 1 mM EDTA,  25 ˚C | 380/460 | (3) |
| Thermolysin (Promega) | Mca-PLGL-Dpa-AR-NH2  (Boc Science) | 2 x 10^-3^ | 50 mM Tris-HCl pH 7.5, 1 mM ZnCl_2,_  25 ˚C | 328/393 | (4) |

**Table S2: Protein concentration of mussel extracts.**

| **Mussel extracts** | **Protein concentration (mg/mL)** |
| --- | --- |
| *D. polymorpha* crude | 4.48 ± 0.08 |
| *D. polymorpha* clarified | 1.76 ± 0.005 |
| *L. costata* (Gill) | 0.89 ± 0.01 |
| *L. costata* (Mantle) | 1.21 ± 0.04 |
| *L. costata* (Foot) | 0.32 ± 0.02 |

**Table S3: Extract combinations used in quantitative proteomics comparisons.** Number of proteins within each combination represented within the respective columns.

| **Combination** | ***L. costata* (Foot)** | ***L. costata* (Gill)** | ***L. costata* (Mantle)** | ***D. polymorpha* crude** | ***D. polymorpha* clarified** |
| --- | --- | --- | --- | --- | --- |
| A-B | 2 | 2 | 0 | 0 | 0 |
| A-B-C | 26 | 26 | 26 | 0 | 0 |
| A-B-C-D | 4 | 4 | 4 | 4 | 0 |
| A-B-C-D-E | 74 | 74 | 74 | 74 | 74 |
| A-B-C-E | 3 | 3 | 3 | 0 | 3 |
| A-B-D-E | 8 | 8 | 0 | 8 | 8 |
| A-C-D-E | 13 | 0 | 13 | 13 | 13 |
| A-D-E | 21 | 0 | 0 | 21 | 21 |
| A-E | 1 | 0 | 0 | 0 | 1 |
| B | 0 | 1 | 0 | 0 | 0 |
| B-C | 0 | 3 | 3 | 0 | 0 |
| B-C-D-E | 0 | 14 | 14 | 14 | 14 |
| B-C-E | 0 | 2 | 2 | 0 | 2 |
| B-D- | 0 | 1 | 0 | 1 | 0 |
| B-D-E | 0 | 34 | 0 | 34 | 34 |
| B-E | 0 | 1 | 0 | 0 | 1 |
| C-D-E | 0 | 0 | 16 | 16 | 16 |
| C-E | 0 | 0 | 1 | 0 | 1 |
| D | 0 | 0 | 0 | 17 | 0 |
| D-E | 0 | 0 | 0 | 316 | 316 |
| E | 0 | 0 | 0 | 0 | 17 |

**Table S4: Protein hits obtained from quantitative proteomics on fraction 18 of clarified extracts of *D. polymorpha.*** See corresponding excel sheet.

*
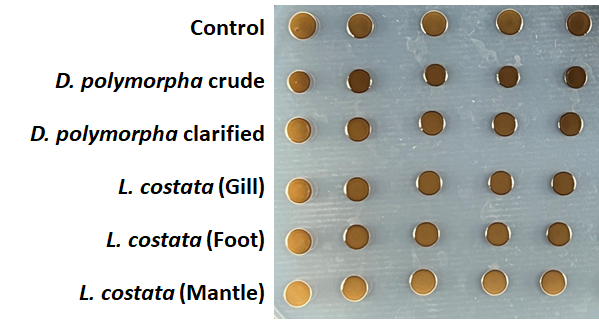
*

**Figure S1: Effect of protein extracts on melanin production in *C. neoformans* H99*.*** Images show *C. neoformans* growth in agar plates supplemented with L-DOPA after 72 hours of incubation with protein extracts at 37 °C.


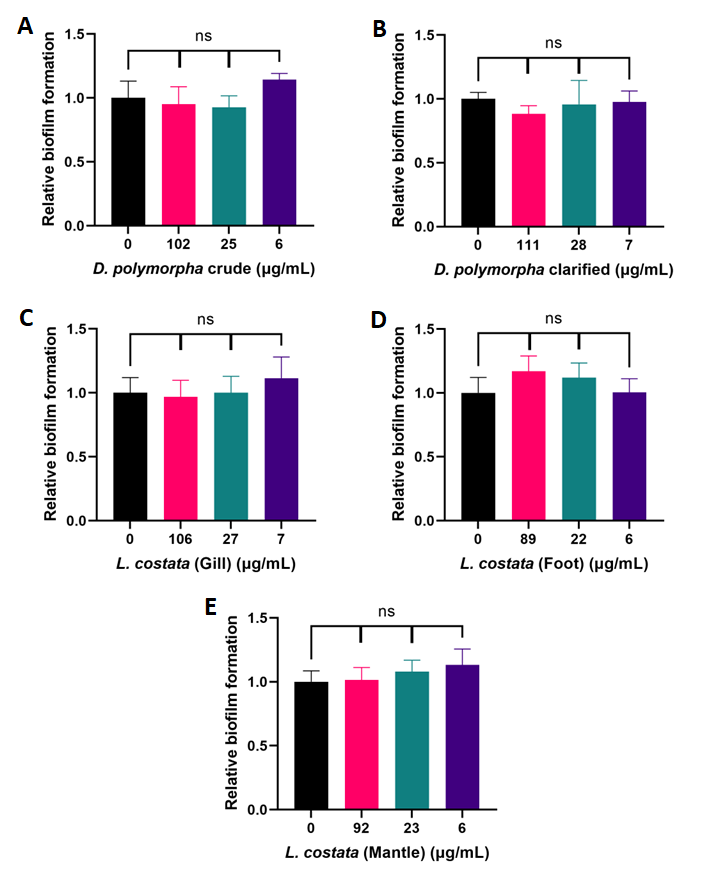


**Figure S2:** **Effect of mussel extracts on pre-established biofilms of *C. neoformans* H99. A**: *D. polymorpha* crude; **B**: *D. polymorpha* clarified; **C:** Crude *L. costata* (Gill); **D:** Crude *L. costata* (Foot) and **E:** Crude *L. costata* (Mantle). Biofilm formation on each condition was normalized to the control without any extract (0 µg/mL). Experiments were performed in biological quadruplicate and technical duplicate. Error bars indicate standard deviation. Statistical analysis was performed using a one-way ANOVA and Dunnett’s multiple comparison tests with a p-value of 0.05. **: p<0.01; ***: p<0.001 and ****: p<0.0001. Figures were created using GraphPad Prism 9.


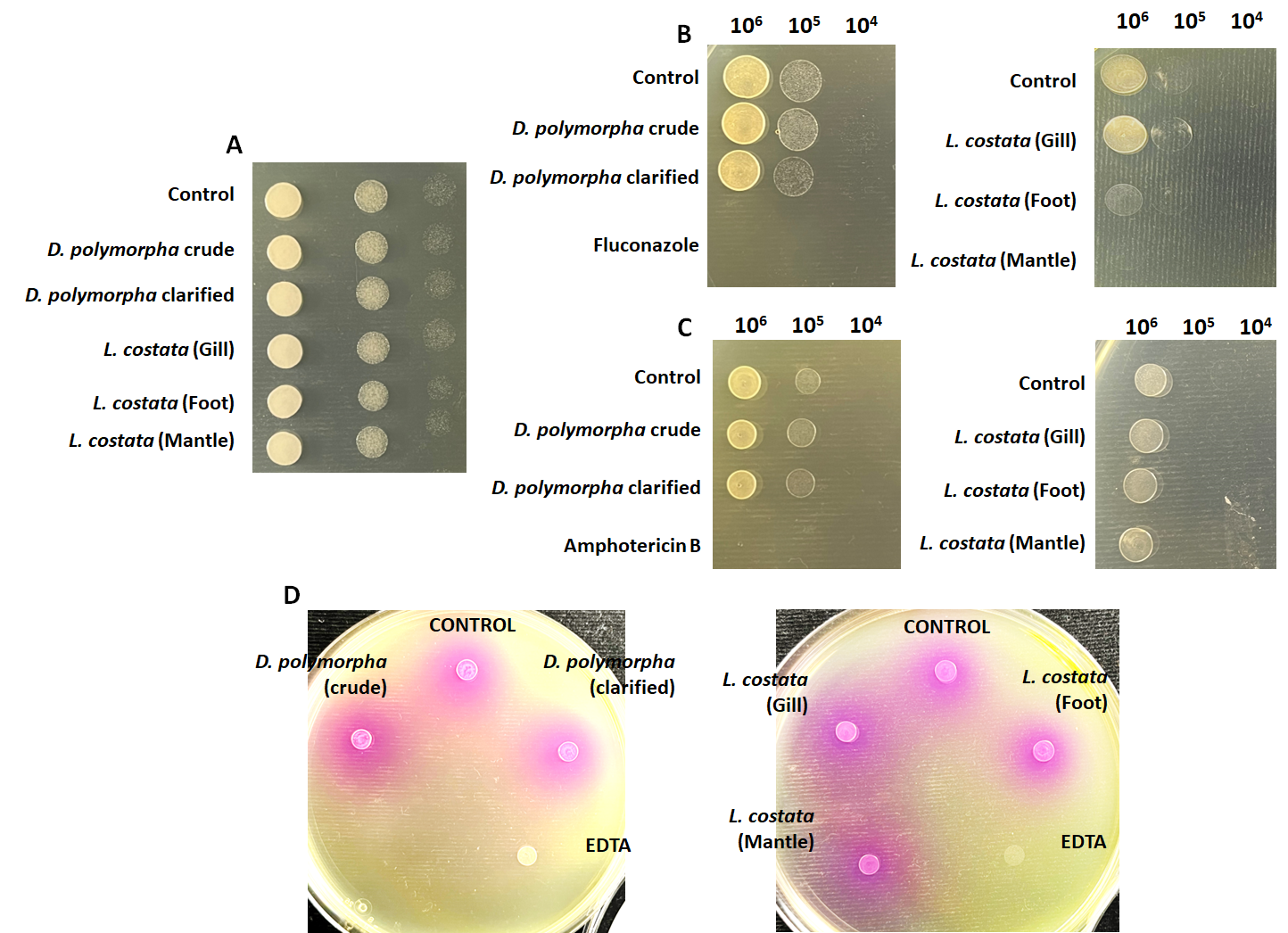


**Figure S3:** **Effect of protein extracts on stress resistance and urease production in *C. neoformans*.** Images show *C. neoformans* growth in agar plates supplemented with different stressors after 4 hours of incubation with protein extracts. **A:** YPD-agar plates. **B:** Membrane stress was performed with SDS 0.01%. Non-treated cells (Control) and Fluconazole (8 µg/mL) were included as positive and negative control, respectively. **C:** Osmotic stress was performed with NaCl 1.5M. Non-treated cells (Control) and Amphotericin B (10 µg/mL) were included as positive and negative control, respectively. Images were taken after 48 h incubation at 37 °C. **D:** Christensen’s Urea Agar after 4 h incubation with protein extracts. Non-treated cells (Control) and EDTA (1 mM) were included as positive and negative control, respectively. Images were taken after 24 h incubation at 37 °C.

**
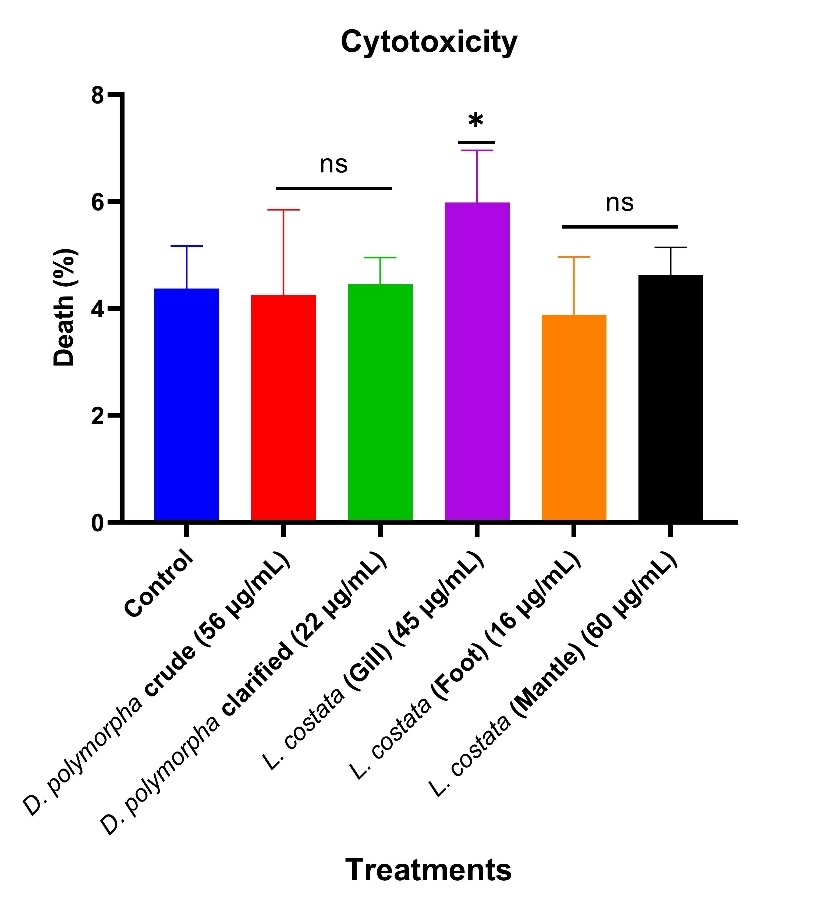
**

**Figure S4:** **Cytotoxicity of protein extracts against BALB/c macrophages.** Bars show a percentage (%) of dead macrophages after 4 h of incubation with protein extracts from mussels. Control indicates natural death after the incubation period. Error bars indicate standard deviation. Statistical analysis was performed using a one-way ANOVA and Dunnett’s multiple comparison tests with a p-value of 0.05. *: p<0.05. The experiment was performed using 6 biological and two technical replicates. Figures were created using GraphPad Prism 9.

References:

1. Kondo H, Shibano Y, Amachi T, Cronin N, Oda K, Dunn BM. 1998. Substrate specificities and kinetic properties of proteinase A from the yeast *Saccharomyces cerevisiae* and the development of a novel substrate. Journal of Biochemistry 124:141–147.

2. Wells JA, Cunningham BC, Graycar TP, Estell DA. 1987. Recruitment of substrate-specificity properties from one enzyme into a related one by protein engineering. Proceedings of the National Academy of Sciences of the United States of America 84:5167–5171.

3. Redzynia I, Ljunggren A, Bujacz A, Abrahamson M, Jaskolski M, Bujacz G. 2009. The crystal structure of the parasite inhibitor chagasin in complex with papain allows identification of structural requirements for broad reactivity and specificity determinants for target proteases. The FEBS journal 276:793–806.

4. Marguerre AK, Krämer R. 2009. Lanthanide-based fluorogenic peptide substrate for the highly sensitive detection of thermolysin. Bioorganic and Medicinal Chemistry Letters 19:5757–5759.
